# Progress in Antibacterial Hydrogel Dressing

**DOI:** 10.3390/gels8080503

**Published:** 2022-08-12

**Authors:** Jie Liu, Wenqi Jiang, Qianyue Xu, Yongjie Zheng

**Affiliations:** 1 College of Light Industry and Textile, Qiqihar University, Qiqihar 161006, China; 2 Engineering Research Center for Hemp and Product in Cold Region of Ministry of Education, Qiqihar 161006, China

**Keywords:** wound dressing, antibacterial mode, antibacterial mechanism, antibacterial properties, hydrogel

## Abstract

Antibacterial hydrogel has excellent antibacterial property and good biocompatibility, water absorption and water retention, swelling, high oxygen permeability, etc.; therefore, it widely applied in biomedicine, intelligent textiles, cosmetics, and other fields, especially for medical dressing. As a wound dressing, the antibacterial hydrogel has the characteristics of absorbing wound liquid, controlling drug release, being non-toxic, being without side effects, and not causing secondary injury to the wound. Its preparation method is simple, and can crosslink via covalent or non-covalent bond, such as γ-radiation croFsslinking, free radical polymerization, graft copolymerization, etc. The raw materials are easy to obtain; usually these include chondroitin sulfate, sodium alginate, polyvinyl alcohol, etc., with different raw materials being used for different antibacterial modes. According to the hydrogel matrix and antibacterial mode, the preparation method, performance, antibacterial mechanism, and classification of antibacterial hydrogels are summarized in this paper, and the future development direction of the antibacterial hydrogel as wound dressing is proposed.

## 1. Introduction

Human skin is an effective barrier to protect the subcutaneous tissue from microbial penetration, and dressing is a temporary substitute skin barrier function of medical textiles. Trauma to the skin can cause many reactions, including increased metabolism, decreased body temperature, excessive loss of water and protein, and imbalance of the endocrine and immune systems. Because of the different causes of wound formation, wound size, shape, exudate volume, and other aspects also vary; therefore, wound dressing requirements are also different [1]. The traditional textile dressing, though its price is lower, has no practical anti-infection function, is dry and easily adheres to the wound, and is not conducive to the anti-infection wound exudate absorption and healing. Most traditional dressings have only a single biological activity, which cannot be applied quickly and efficiently to treat different wounds [2]. Wounds can be acute or chronic. An acute wound is a wound that forms suddenly and will heal soon; a chronic injury does not heal as quickly as expected and usually takes one to three months or longer to heal [3]. The main reason for the failure of chronic wounds to heal quickly is the accumulation of microorganisms in the wound bed. Chronic wound infections can lead to complications such as amputation, sepsis, and even death [4]. Wound stent dressings can treat chronic wounds, such as those caused by dermal injuries or burns, which can help reduce scar formation [5]. There are mainly 3–10 dominant microorganisms in the wound, including *Staphylococcus aureus*, *Streptococcus*, *Pseudomonas*, anaerobic bacteria, etc. In addition, there are hundreds of microorganisms of different species attached and embedded in the extracellular matrix of biofilm [6]. The ideal dressing should be non-toxic, have no side effects, be moisturizing and absorbent, and promote healing without causing secondary injury, rather than merely acting as a protective barrier [7,8,9]. Therefore, the development of new medical dressings has gradually become more popular. Various wound dressings have been studied, such as rubber [10,11,12], polyionic liquid membrane [13,14], electrostatically spun nanofibers [15,16,17], and hydrogel [18]. Among these dressings, a hydrogel is the material that best meets people’s requirements for ideal dressings [19]. In 1960, Wichterle and Lim successfully prepared a hydrogel by polymerizing methyl-2-hydroxyethyl methacrylate and made the first contact lens in history [20], thus starting the application research on hydrogels.

Hydrogel is a kind of polymer material with a three-dimensional network structure, which is composed of polymer chains crosslinked through physical or covalent bonds [21,22]. Since they contain a large number of hydrophilic groups, hydrogels can absorb tens or even hundreds of times their own mass of water; they are of high hydrophilicity, display good swelling in water, and have good water retention [23]. Due to high water content, hydrogels can cool the wound and reduce the warm feeling associated with the inflamed tissue [24]. With good biocompatibility and cell adhesion, hydrogels can directly contact the injury, reduce the loss of body fluid, and prevent secondary infection injury. They can be used locally to help wound healing [25,26,27]. Hydrogels can also be coated on catheters, central venous catheters, joint implants, and dental implants for drug release [28,29,30]. By grafting hydrogel monomer onto fibers, coating hydrogel onto fabrics, intelligent textiles, or textile dressings, and printing and dyeing, wastewater adsorption materials can be made [31,32]. Due to a series of excellent properties, hydrogels have received much attention, especially in biomedical fields [33,34]. However, with the abuse of traditional hydrogels, bacteria become resistant to antibiotics, and conventional hydrogels gradually lose their advantages. To solve these problems, antibacterial hydrogels, which have the dual functions of hydrogel and antibacterial, were found. The preparation process of antibacterial hydrogels is simple and the structure is diverse, which attracts attention, and a variety of advanced antibacterial hydrogels have been developed successively.

Antibacterial hydrogel has been widely used in many fields, such as biomedicine and intelligent textiles, and it is one of the suitable biological materials for drug delivery in the area of antibacterial treatment. Antibacterial hydrogels have also become a new focus of research, and their research and application have also begun to develop rapidly. Various preparation methods of antibacterial hydrogels have been widely used in biomedical, tissue-engineering, and intelligent textiles, such as drug delivery, regenerative medicine, trauma dressing, vitro diagnosis, etc. [35]. As a medical dressing, an antibacterial hydrogel can have multiple properties simultaneously. The most important property is bacteriostatic or antibacterial. The antibacterial spectrum of antibacterial hydrogels is determined by the antibacterial components carried, and various materials have different antibacterial properties against different strains [36]. There are many methods for testing antibacterial properties of antibacterial hydrogels, such as the bacteriostatic circle method, oscillation method, plate colony counting method, OD counting method, scanning electron microscopy method, etc. Among them, the bacteriostatic circle method is commonly used because of its simple operation and intuitive results [37,38]. For example, the silver nanoparticles doped conductive polymer hydrogel system (Ag-NPs/CPH) can be used as a dressing, which has good electrical conductivity, biocompatibility, mechanical properties, and antibacterial properties, and can be used to heal seriously infected injuries [39]. TiO_2_ combined with nanoparticles also has bacteriostatic properties. Khashan et al. prepared TiO_2_ nanoparticles by liquid laser ablation, which has excellent bacteriostatic properties, and can be applied in hydrogels to better inhibit bacteria [40]. After the copolymer of methylacryamide (MA Ade) and 3-dimethyl (methylacryl oxyethyl) ammonium propane sulfonate (DMAPS) was composed, and chitosan was introduced and the synthesized PDMAPS-co-PMA-Ade/chitosan hydrogel was used as dressing with antibacterial activity and repeated adhesion ability. Wang et al. synthesized the polyionic liquid antibacterial hydrogel PMAV. They have high protein adsorption properties, good mechanical properties, and biocompatibility. They can be used repeatedly and maintain good antibacterial properties. They are available for reuse as non-releasing antibacterial hydrogel dressings [41,42].

To prevent the infection of pathogenic microorganisms in vitro, the use of antibacterial hydrogel dressing directly in contact with human tissue can effectively isolate microorganisms and maintain oxygen to the wound site in order to aid the wound in healing as soon as possible. This paper summarizes the mechanism, performance, and preparation of antibacterial hydrogels as wound dressing, and finally, prospects for the development of antibacterial hydrogels.

## 2. Preparation of Antibacterial Hydrogels

The raw material of antibacterial hydrogel for dressing mainly consists of three parts, namely the monomer, initiator, and crosslinking agent. In general, hydrogels are prepared by hydrophilic monomers. In some exceptional cases, hydrophobic monomers can be added to adjust some specific application properties of hydrogels.

There are various methods for preparing antibacterial hydrogels, such as the radiation method, chemical crosslinking, physical crosslinking, polymerization grafting, free radical crosslinking polymerization, chemical–physical crosslinking, and so on. The raw materials used are different, the preparation methods are other, and the strains inhibited are other, but all of them have significant antibacterial effects. The preparation methods, materials, antibacterial ability, and application of various antibacterial hydrogels are shown in Table 1.

**Figure 1:**
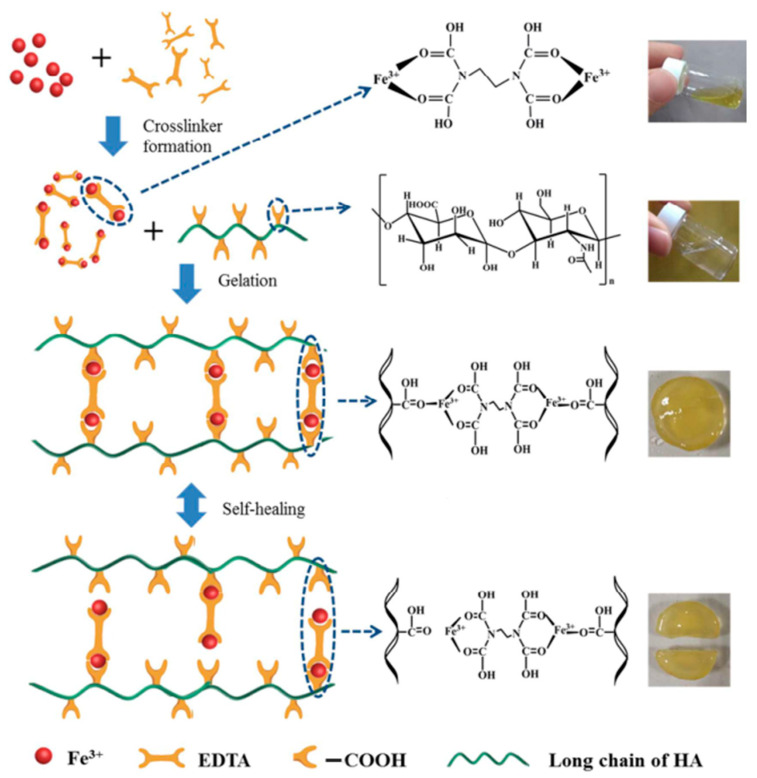
Preparation and self-healing mechanism of HA-Fe-EDTA hydrogel. Reproduced with permission [44].

Physical crosslinking is formed by non-covalent crosslinking, such as hydrogen bonds, interactions between hydrophobic groups, ion complexation, electrostatic interactions, etc. Hydrogels formed by physical crosslinking are quickly dissolved when environmental conditions such as temperature, pH value, and the ionic strength of solution change [43]. For example, Tian et al. used sodium ferric diamine tetraacetate (EDTA-Fe^3+^) as a crosslinking agent, physically crosslinking with hyaluronic acid (HA), and a hydrogel that can be triggered by bacteria at the site of infection and can quickly self-heal was developed. The dynamic ionic bond between EDTA-Fe^3+^ and HA can rapidly reestablish within minutes. When used as a dressing, HA can degrade at the site of infection, and hydrogels can release a Fe^3+^ complex locally. The complex is quickly adsorbed by surrounding bacteria and reduced to Fe^2+^, which produces hydrogen peroxide (H_2_O_2_) with bacteria and inflammatory cells. It forms hydroxyl free radicals through the Fenton reaction to destroy the bacterial structure. It can effectively kill *Escherichia coli* and *Staphylococcus aureus*, promote angiogenesis, and create new skin within ten days. The formation and self-healing mechanism of the HA-Fe-EDTA hydrogel is shown in Figure 1 [44].

**Figure 2:**
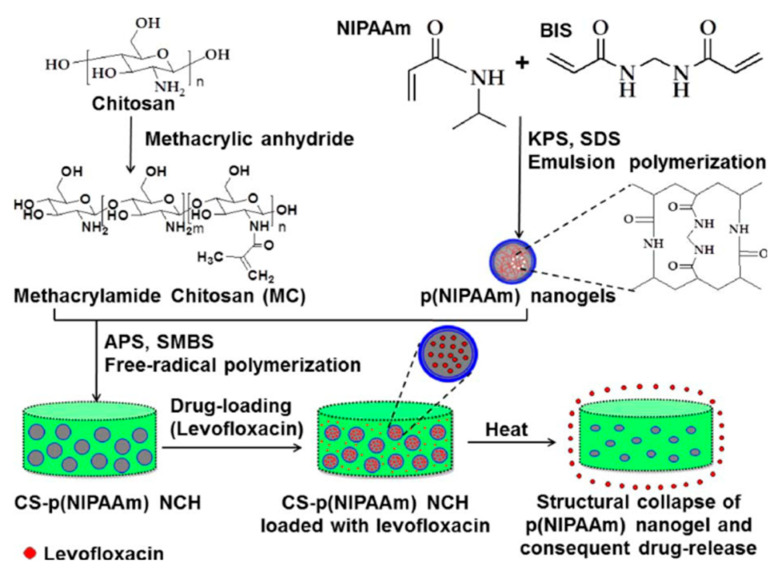
Preparation of CS-p(NIPAAm) nanocomposite hydrogel. Reproduced with permission [47].

Chemical crosslinking hydrogels are made by covalent crosslinking, and polymer chains are covalently bonded by crosslinking agents [45]. Hydrogels formed by chemical cross-linking are relatively stable. For example, Li et al. studied the preparation of polyurethane/polyvinyl alcohol hydrogels by chemical crosslinking and in situ synthesis. Adding silver particles to the hydrogel can improve the antibacterial activity of the hydrogel; improve Young’s modulus, tensile strength, and elongation; and maintain the water absorption ability good biocompatibility of the hydrogel, which is an excellent choice for wound dressing [46]. Shin et al. prepared thermoresponsive nanocomposite hydrogels by combining nanostructured particles with 3D hydrogels. The preparation of CS-p(NIPAAm) nanocomposite hydrogel is shown in Figure 2. Poly(*N*-isopropyl acrylamide) [P(NIPAAm)] nanocomposite hydrogels were synthesized by emulsion polymerization and introduced into methyl acrylamide CS (MC) solution. They were then embedded into the hydrogel for free radical-induced crosslinking reaction. Finally, levofloxacin (LFX) was loaded into the nanocomposite hydrogel to enhance the drug loading ability of the hydrogel [47].

**Figure 3:**
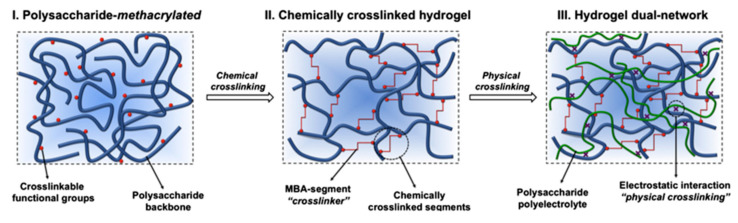
Schematic representation of the formation of a double network hydrogel (i. Polysaccharide-methacrylated; ii. Chemically crosslinked hydrogel; iii. Hydrogel dual-network). Reproduced with permission [48].

Chemical–physical crosslinking. Fajardo et al. used K_2_S_2_O_8_ as an initiator and glycidyl methacrylate (GMA), chitosan (CHT), or chondroitin sulfate (CS) as raw materials to synthesize polymers. Then, chemical hydrogels (CHT-gel and CS-gel) were prepared by chemical crosslinking using *N*,*N*-methylene bisacrylamide (MBA) as a cross-linking agent. The two chemical hydrogels were then immersed into the CS or CHT stock solution for physical crosslinking. Due to the polyelectrolyte complexation between CHT and CS chains, the dual network antibacterial hydrogels (CHT-gel/CS and CS-gel/CHT) were prepared. The formation of a double network hydrogel is shown in Figure 3.

Radiation crosslinking refers to the technical means of a crosslinking reaction between long polymer chains caused by radiation. Alcantara et al. prepared antibacterial hydrogels using poly(*N*-vinyl-2-pyrrolidone) (PVP) and polyvinyl alcohol (PVA) by the method of radiation crosslinking. The aqueous solution containing AgNO_3_ was added to PVP and PVA by γ-ray irradiation with a 60 Co source, which could realize the crosslinking of polymers and the synthesis of AgNPs simultaneously. PVP/AgNPs hydrogel has antibacterial properties against *Pseudomonas aeruginosa* and *Staphylococcus aureus*. PVA/AgNPs hydrogel has antibacterial properties against *Pseudomonas aeruginosa* and antibacterial activity against *Staphylococcus aureus*. Two antibacterial hydrogels can be used as dressings to treat common wounds and burns [49].

In a free radical polymerization reaction, the monomer molecules are activated into active free radicals under the action of light, heat, radiation, and initiator, then linked with the monomer polymerization to form polymers. Wang et al. effectively prepared CMO-loaded composite emulsion hydrogel by acrylamide radical polymerization using oil-in-water concentrated emulsion as a continuous phase. The prepared CMO-Loaded composite hydrogel has good long-term antibacterial activity against Staphylococcus aureus and *Escherichia coli* [50].

Graft copolymerization is a reaction in which a macromolecular chain binds an appropriate branch or functional side group by chemical bonding. Grafting modification of polymer is an effective method to improve the properties of polymer materials. The supernormal transition metals Ce (IV) and Cu (III), which react with −OH groups, can produce free radicals to initiate vinyl polymerization. Ma et al. prepared antimicrobial hydrogels by grafting polymers on the surface of Heloxite nanotubes (HNT) with dimethyl aminoethyl methacrylate (DMAEMA) and sodium acrylate (AA-NA) using a REDOX system mediated by supernormal transition metals (Ce(IV) and Cu(III)) in aqueous medium at 35 °C. The prepared polycationic grafted nanotubes and polyanionic grafted nanotubes can be mixed in an aqueous medium to form hydrogels, and the prepared hydrogels have an antibacterial effect on *Escherichia coli* [51].

**Table 1:** Preparation methods, properties, and application of various antibacterial hydrogels.

Synthetic Methods	Species of Hydrogels	Materials	Antimicrobial Capability	Application	Ref.
Chemical crosslinking	Acacia gum-PVA hydrogel	Acacia gum, PVA, glutaraldehyde, salicylic	Against *Bacillus subtilis*, *P.* *aeruginosa*, *E. coli* and *S. aureus*	Wound dressing	[52]
	Silk fibroin crosslinked glycyrrhizic acid and silver sydrogels	SF, Ag, GA	Against *S. aureus*, *P. aeruginosa*		[53]
	PHCI hydrogel	1,3-dibromo-2-propanol, trans-1,4-cyclohexanediamine	Can adsorb and kill *S.aureus* and *E.coli* electrostatically		[54]
	Silk fibroin/chitosan hydrogel	SF, CS, LiBr	Against *Bacillus subtilis*, methicillin-resistant *S. aureus*, and *E. coli* strains with a contact-killing efficacy of 100%		[55]
Physical crosslinking	Antibacterial chitosan/silver bio-nanocomposite	STPP, chitosan, AgNPs	The antibacterial activity against *E. coli* and *S. aureus* lasted for 1 week.	Drug carrier	[56]
	Polysaccharide based physically crosslinked double-network antibacterial hydrogel	SA, CS, Zn^2+^	Against *E. coli* and *S. aureus*	Biomedicineureus fields	[57]
	AA-Al^3+^-MGA-[VBIm]Br hydrogel	AA, 1-vinyl-3-butylimidazolium, COOH-modified gum arabic, AlCl_3_	Against *E. coli*, *S. aureus*, and *C. albicans*.	Wound dressing	[58]
	PVA-TA hydrogel	PVA, TA	Against *E. coli* and *S. aureus*	Biomedical fields	[59]
Freezing-thawing cycles	Nano-TiO_2_/CMCS/PVA composite hydrogel	PVA, CMCS, Nano-TiO_2_	Against *E. coli* and *S. aureus*	Cosmetics, medical dressings	[60]
	AgNPs and PVA/CH hydrogel	AgNPs, PVA, CH	Against gram + ve and gram − ve bacteria	Wound dressing	[61]
	A polyvinyl alcohol (PVA) hydrogel loaded with guava leaf extract (GLE)	GLE, PVA	Against *S. aureus* and *P. aeruginosa*.		[62]
Uv crosslinking method	PVA-SbQ/MMT composite hydrogel	MMT, PVA-SbQ	against *S. aureus* was up to 99.95%.	Wound dressing	[63]
Solution polymerization	ZnO@GDM hydrogel	ZnO, GelMA, DMAA, MAA	Against *E.coli* reached more than 98%	Biomedical fields	[64]
	Poly(DMA-co-AAc) hydrogel	DMA, AAc, ammonium persulfate	Inhibit the growth of *S. aureus*.	Antibacterial materials	[65]
Photoinduction	Hydrogel containing silver nanoparticles	AgNO_3_, MDEA, acrylamide, bis-AAm	Fully inhibition of the growth of *Acinetobacter johnsonii* and *E. coli*.	Wound dressing	[66]
	Antibacterial acrylamide hydrogels containing silver	Acrylamide, silver nitrate, trisodium citrate dihydrate,1-[4-(2-hydroxy-ethoxy)phenyl]-2- hydroxy-2-methyl-1-propane-1-one	Against pathogenic *E. coli* O157:H7, *S. aureus*, and non-pathogenic *E. coli* K-12	Water-based applications	[67]
	Chitosan-PEG hydrogels	Chitosan derivatives, PEG	100% inhibition of the *E. coli* and *S. aureus*	Bio-functional materials	[68]
Coacervation	CS@CMC@ZeoliteP@KDF hydrogel	ZeoliteP, KDF, CMC, CS	Inhibit the growth of *E.coli* and *S. aureus*.	Bacteriostatic agent	[69]
Chemical–physical crosslinking	DR-CC hydrogel	Carboxylated chitosan, diazoresin	Can kill *E. coli* and *S.aureus*.	Wound dressing	[70]
	β-CD/PEI/PVA composite hydrogels	β-CD, PVA, PEI	Against *E. coli* and *S. aureus*		[71]
	Lignin hydrogels	SBMA, lignin-MA	Antimicrobial performance of 94.8% reduction of *E. coli* and 95.7% of *S. aureus*.	Biomedical fields	[72]
	Chitosan/PV A-based hydrogels	CS, PVA	Against *S.aureus* and *K. pneumonia*	Oral dressing	[73]
Free radical crosslinking polymerization	P(MMA-co-MAA)/Ag nanocomposite	MMA, MAA, AgNPs	Against *S. aureus* and *B. subtilis*.	Smart material	[74]
	Poly(N-[3-(dimethylaminopropyl)] methacrylamide) hydrogels	Cetyltrimethylammonium bromide, KPS, BIS	Against *E. coli*	Antimicrobial agent	[75]
	Tea Ag nanocomposite hydrogels	Acrylamide, MBA, TEMDA, KPS	Against *E. coli* and *S. aureus*.	Wound dressing	[76]
	D/SD-g-PAA-based hydrogels	MBA, AA	Can kill *S. aureus* and *E. coli* and *Klebsiella* spp.		[77]
Gamma-ray irradiation	Metronidazole/poly(acrylic acid) hydrogel	Metronidazole, poly(acrylic acid)	Against *E. coli*, *S. aureus*, *S. mutans*.	Wound dressing	[78]
	P-PVA hydrogel	phosphorus-containing PVA	Against various fungi and bacteria	Biological fields	[79]
	Poly(Agar-co-AAc) hydrogels	AAc, agar	Against *E. coli* and *S. aureus*.	Wound dressing	[80]
	Polyvinyl pyrrolidone/carboxymethyl cellulose hydrogels	Polyvinyl pyrrolidone, carboxymethyl cellulose, AgNPs	Against bacterial, *S. aureus*, *P. aeruginosa*, *E. coli* and *Candida albicans*	Therapeutic dressing	[81]
Reverse emulsification- diffusion	Alginate nano hydrogel	Alginate, iron (II)-chloride, sodium lactate	Against *E. coli* and *S. aureus*.	Smart textile	[82]

## 3. Performance of Antibacterial Hydrogels

As an ideal medical dressing, antibacterial hydrogel should have the following properties: excellent antibacterial performance, high permeability, good biocompatibility, and water absorption capacity.

### 3.1. Antibacterial Properties

As a medical dressing, antibacterial performance is the primary performance that a hydrogel must have. It should have an antibacterial or bacteriostatic effect, inhibit the breeding of harmful microorganisms, and resist wound infection [83]. The antibacterial effect is mainly tested by the bacteriostatic zone method; that is, the bacteriostatic zone diagram and diameter comparison of hydrogel against *Escherichia coli* (typical Gram-negative bacteria) and *Staphylococcus aureus* (specific Gram-positive bacteria) were tested via an in vitro antibacterial experiment. For example, Gharibi et al. prepared an antibacterial hydrogel containing torymic, Si-CAQ (3-glycidyloxypropyl) trimethoxysilane, and poly(vinyl alcohol) for wound dressing. The hydrogel has good antibacterial activity against *Bacillus subtilis*, METHicillin-resistant *Staphylococcus aureus*, and *Escherichia coli*, and the bactericidal effect is 100%. It can prevent the development of infection at the injured site and promote wound healing [84]. Sun et al. prepared sodium alginate double crosslinking hydrogel fibers loaded with sulfadiazine and crosslinked with calcium ion and glutaraldehyde. The sodium alginate hydrogel fibers had good mechanical strength, biocompatibility, and sustainable drug release, and the antibacterial test showed that the antibacterial hydrogel could kill 99.9% of *Staphylococcus aureus* and *Escherichia coli* [85].

**Figure 4:**
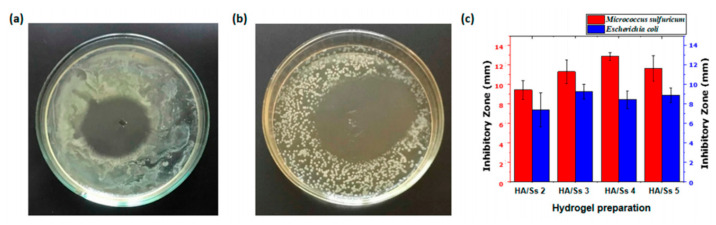
Antibacterial properties of hyaluronic acid/spider silk-based hydrogels. (**a**) Inhibitory band test of hyaluronic acid/spider silk-based hydrogels on gram negative *Escherichia coli* bacteria. (**b**) Inhibitory band test of hyaluronic acid/spider silk-based hydrogels on gram positive *Micrococcus sulfuricum* bacteria. (**c**) Inhibitory zone length of hyaluronic acid/spider silk-based hydrogels on gram negative (*Escherichia coli*) and gram positive (*Micrococcus sulfuricum*) bacteria. Reproduced with permission [86].

Withanage et al. prepared (hyaluronic acid)/(spider silk) hydrogels by the method of chemical crosslinking. Antibacterial properties of hyaluronic acid/spider silk-based hydrogels are shown in Figure 4. The water absorption capacity of the hydrogel can reach 15–30 times its weight and has vigorous antibacterial activity against Gram-negative and Gram-positive bacteria. It is suitable for wound dressings, prosthesis implant coatings, and catheter coatings [86].

### 3.2. Water Absorption and Swelling Capacity

**Figure 5:**
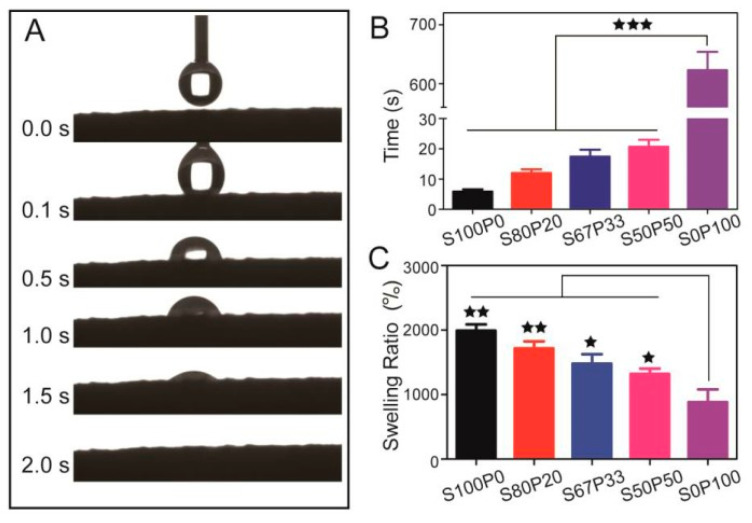
Water absorption and swelling behavior of SS/PVA gels. (**A**) Water contact angle of S50/P50; (**B**) Time required for SS/PVA gels to achieve swelling equilibrium; (**C**) Swelling ratio of SS/PVA gels after immersion into PBS buffers (pH 4, 7.4, 10) at 37 °C. *n* = 3 per group; * *p* < 0.05, ** *p* < 0.01, *** *p* < 0.001. Reproduced with permission [89].

Because they contain a large number of hydrophilic groups, hydrogels have intense swelling and water absorption capacity. Swelling occurs when the gel absorbs water, which is crucial for the gel, because it affects the spread of the bioactive agent and drug that is trapped. When applied in wound dressings, they can absorb tissue fluid or blood from the wound, keep the wound surface moist, and prevent the loss of tissue fluid. Generally, the water absorption properties of treated materials are studied by moisture regain, water retention, wet fabric drying time at room temperature, and vertical core wicking test. For example, Zhang et al. prepared an antibacterial wound dressing by loading antibacterial drugs onto aramid nanofiber (ANFs) hydrogel. The water content of ANFs hydrogels is more than 98 percent, they have high water adsorption properties (>10,000%) and good water retention ability (water retention of more than 5000 percent after incubating at RH 30% for eight h), and they are capable of absorbing and retaining the wound exudate to form a moist environment [87]. Khan et al. developed a chitosan-based antibacterial hydrogel, which has a swelling ratio of 1036%; anda water absorption ratio of more than ten times [88]. Gang et al. designed a sericin/poly(vinyl alcohol) hydrogel as a drug delivery carrier for wound dressing; its water absorption and swelling behavior are shown in Figure 5.

Figure 5A shows that SS/PVA (S50P50) hydrogel absorbed water droplets in 2 s and had super hydrophilicity. Figure 5B shows the time required for different SS/PVA hydrogels to reach swelling equilibrium. The swelling equilibrium time of S50P50 is 20 s, indicating that its hydrophilicity is good. Figure 5C shows sericin and SS/PVA hydrogels had swelling ratios of 1200–1800 percent, whereas that of PVA had 800 percent, indicating SS/PVA hydrogel had the best swelling ability.

### 3.3. Drug Release Properties

The unique network structure makes antibacterial hydrogel very suitable for drug carriers. When used in wound dressings, it has excellent drug release performance, that is, the ability to continuously release antibacterial drugs, which can be sustained for a long time to promote wound healing, and can avoid frequent dressing changes, thus reducing the risk of wound overexposure to bacteria [90,91]. Drug release properties can generally be analyzed by measuring in vitro release time or cumulative drug release over a certain period. For example, Erdagi studied the neomycin release performance of the crosslinking gelatin/DGN-NC-based hydrogels; the results showed a rapid release within the initial 15 min and then sustained release for 9–12 h. The material is suitable for a polymer antibiotic matrix in wound dressings [92]. Ak. Prepared poly(vinyl alcohol) chitosan-loaded AgNPs hydrogel. They showed that the maximum release amount of the hydrogel was 91.83%, and the release time of the concentration-dependent inhibition zone (ZOI) was over 24 h, respectively [93]. Guan et al. prepared Ag/AgO/carboxymethyl chitosan antibacterial hydrogel, with a cumulative drug release of 75.20% within 12 h and a maximum inhibition rate of 92.32% on *E. coli* [94]. It is an effective method of drug delivery.

### 3.4. Permeability

To prevent the growth of anaerobic bacteria, leading to wound infection and inflammation, antibacterial hydrogel dressings need to have specific air permeability. Singer et al. developed a hydrogel wound dressing for drug delivery using polymerization of polysaccharide gum. The prepared hydrogel membrane was permeable to O_2_ and H_2_O vapors, but impervious to microorganisms, and can protect the wound surface from bacterial invasion [95]. Wang studied the application of Chitosan-graft-poly(*N*-isopropyl acrylamide) (CS-g-PNIPAAm) microgels in a water vapor-permeable fabric. It can meet the requirement of high water vapor permeability at high temperatures [96]. Yan prepared methyl acrylamide adenine and 3-(methyl acryloxyethyl)-dimethyl-ammonium propane sulfonate copolymerized hydrogels, and loaded chitosan to prepare semi-interpenetrating polymer network hydrogels (PDM/C). The WVTR of band-aid, gauze, and PDM/C hydrogel were 2399 ± 524, 2045 ± 131, and 781 ± 148 g/m^2^/day, respectively. The WVTR of PDM/C hydrogel was much smaller than that of the other two wound dressings, indicating that PDM/C hydrogel can effectively reduce water evaporation and keep the wound moist [97].

### 3.5. Biocompatibility

Biocompatibility is an essential performance of antibacterial hydrogels. As dressings, antibacterial hydrogels need to be non-toxic to cells and not toxic to human tissues. Zhu used several reagents including chitosan, glacial acetic acid, nanosilver, and poloxamer to prepare nanosilver antibacterial hydrogel. The antibacterial hydrogel made was found to have good biocompatibility and can effectively promote cell proliferation and differentiation without releasing any toxic substances through biocompatibility tests [98]. Chen et al. synthesized PaA-Cu-MOP hydrogel. After testing, PaA-Cu-MOP hydrogel can promote cell proliferation and blood vessel generation, up-regulate cytokines, and even outperform recombinant human epidermal growth factors. Therefore, PaA-Cu-MOP hydrogel has excellent biocompatibility and can accelerate wound healing [99].

## 4. Classification and Mechanism of Antibacterial Hydrogels

Depending on the antibacterial mode, an antibacterial hydrogel can be divided into five categories: (1) antibacterial hydrogel containing inorganic nanoparticles; (2) antibiotic-loaded antibacterial hydrogel that can directly kill cells to achieve sterilization; (3) hydrogel with inherent antimicrobial activity; (4) photosensitive antibacterial hydrogel; and (5) hydrogels with synergetic effects. Currently, most of the research is on antibacterial hydrogels containing silver nanoparticles, chitosan hydrogels, and antibiotic-loaded hydrogels.

### 4.1. Antibacterial Hydrogels Containing Inorganic Nanoparticles

As antibacterial agents, inorganic nanoparticles have attracted attention due to their broad spectrum of antibacterial activity and non-drug resistance. Metal ions in inorganic nanoparticles and metal nanoparticles show high catalytic activity in the ortho–para proton conversion reaction. Therefore, more attention has been paid to metal ions such as gold (Au), silver (Ag), and copper (Cu). Because the reaction rate of metal particles depends on the properties of the metal: Au > Ag > Cu, among them, silver has a high-cost performance, making silver nanoparticles become a hot research topic [100]. Metal oxides in inorganic nanoparticles can interact with the surface of the material, so metal oxides in the form of nanoparticles, such as titanium dioxide (TiO_2_), zinc oxide (ZnO), nickel oxide (NiO), etc., have also become the leading research object [101].

#### 4.1.1. Antibacterial Hydrogel Containing Silver Nanoparticles

**Figure 6:**
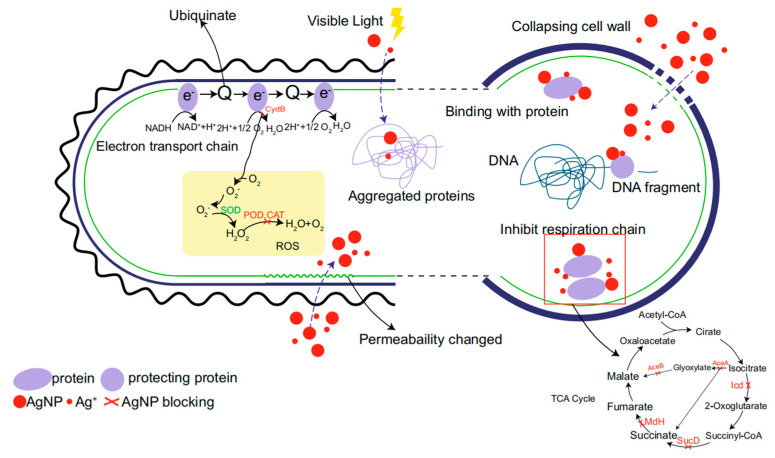
Antibacterial mechanism of Ag NPs. Reproduced with permission [108].

It is well-known that silver nanoparticles (Ag NPs) have potent bactericidal activity against a variety of bacteria. The significant antibacterial effect of Ag NPs is due to their large specific surface area, which increases the contact area between Ag NPs and the bacterial membrane. This induces cytoplasmic leakage to inhibit bacterial growth. In addition, Ag NPs have great binding power and affinity with most macromolecules, and their contact with bacterial membranes will lead to the disintegration and death of bacterial cells. So far, the efficient antibacterial activity of Ag NPs has been confirmed in many studies. Ag NPs have been found to kill many species of bacteria, including *Staphylococcus aureus*, *Pseudomonas aeruginosa*, *Escherichia coli*, *Bacillus subtilis*, *Vibrio cholera*, *Salmonella typhus*, *Enterococcus faecalis*, *Klebsiella* sp., *Listeria* sp., and *Acinetobacter* sp. [102,103,104]. At present, the antibacterial mechanism of Ag NPs is still controversial. Still, its antibacterial mechanism can be summarized into six types: interference with bacterial protein synthesis and folding inhibition of bacterial respiratory chain, induction of bacterial genetic toxicity, induction of photocatalytic destruction of bacterial protein and rupture of the cell membrane, and induction of bacterial oxidative stress reaction to produce reactive oxygen species [105,106,107]. The antibacterial mechanism of silver nanoparticles is shown in Figure 6. Silver ions can disrupt the respiration of bacteria and destroy the cell membrane of bacteria. To bond with the DNA in the cell membrane and to prevent the replication of genetic information of bacterial cells from achieving the bactericidal effect, through research, silver has a more substantial bactericidal effect in the nanometer state.

#### 4.1.2. Antibacterial Hydrogel Containing Zinc Oxide Nanoparticles

Zinc Oxide nanoparticles (ZnO NPs) can kill microorganisms by a variety of mechanisms: (1) ZnO NPs can bind firmly to bacterial cell membranes and destroy lipids and proteins of cell membranes; the result is an increase in membrane permeability, which causes cytoplasmic content to flow out of the cell, leading to cell death. (2) ZnO NPs can also induce the formation of Zn^2+^ and reactive oxygen species (ROS), which can destroy bacterial cells. (3) Small particles of ZnO NPs can increase the membrane permeability and then enter the cytoplasm of bacterial cells, exerting oxidative stress on the cytoplasm and producing substantial toxicity to cells [109,110]. (4) Under ultraviolet radiation, many free radicals are generated on the surface of zinc oxide nanoparticles. When the free radicals contact microorganisms, the organic matter of microorganisms is oxidized into carbon dioxide. Zinc oxide nanoparticles can kill microorganisms in a short time. Hydrogels containing zinc oxide nanoparticles have the potential to facilitate rapid wound healing [111,112]. Majumder et al. prepared the biomimetic composite dressing by grafting hydrogel onto silk fabric and being further coated sonochemical with ZnO NPs to impart these dressing antimicrobial properties [113]. Baghaie et al. successfully prepared a novel synthetic hydrogel film by immobilizing ZnO powder on PVA/starch/chitosan polymer. The results showed that the addition of ZnO NPs could improve the tensile strength of the hydrogel and reduce the porosity of the hydrogel membrane, and the cell viability was over 87%. The wound healing rate in the hydrogel containing ZnO NPs was 96%, while the wound healing rate in the control group was 79% [114].

#### 4.1.3. Antibacterial Hydrogel Containing Titanium Dioxide Nanoparticles

The antibacterial mechanism of titanium dioxide nanoparticles (TiO_2_ NPs) is that TiO_2_ will generate reactive oxygen species (ROS), including hydrogen peroxide (H_2_O_2_) and hydroxyl radical (·OH), after being exposed to near-ultraviolet light in the process of photocatalysis. When TiO_2_ radiated by UV light is close to bacteria, ROS damages the bacterial cell membrane, damages the semi-permeability of the membrane, interferes with and oxidizes the bacterial cell membrane, and achieves the purpose of sterilization. TiO_2_ NPs can kill *Escherichia coli*, *Staphylococcus aureus*, *Pseudomonas aeruginosa*, *Enterococcus faecalis*, *Candida albicans*, and other bacteria [115,116], and TiO_2_ NPs also has a specific destructive effect on viruses. TiO_2_ was used to coordinate with Ag ions to enhance their antibacterial activity. Ghosh et al. synthesized silver-modified titanium dioxide nanoparticles using the green hydrosol-gel method. The product is heat-treated at 450 °C and 600 °C and can achieve a good bactericidal effect. The amount of silver added and the heat treatment process have a significant influence on the phase composition, microstructure, and properties of TiO_2_. This material is suitable for application in healthcare due to its excellent antimicrobial properties, helping to reduce the spread of Gram-negative bacteria such as *E. coli* [117,118].

#### 4.1.4. Antibacterial Hydrogel Containing Other Nanoparticles

In addition to the above-mentioned commonly used nanoparticles, there are several other antibacterial hydrogels containing nanoparticles, such as magnetic nanoparticles, copper nanoparticles, etc. Among them, magnetic nanoparticles have been widely used in biomedicine, nuclear magnetic resonance imaging, and environmental protection, due to their unique physical and chemical properties. The antibacterial mechanism of magnetic nanoparticles is mainly interference with the sulfhydryl group of bacterial proteins and then causing damage to bacteria. Xiong found that magnetic particles showed a particular antibacterial effect on *Escherichia coli*, *Staphylococcus aureus*, *Pseudomonas aeruginosa*, *Salmonella typhi*, *Pasteurella multocida*, and other drug-resistant bacteria. Compared with standard drugs, magnetic nanoparticles showed more substantial antibacterial effects against all bacteria [119]. Magnetic nanoparticles are mostly combined with other metals or metal compounds to achieve the purpose of antibacterial, such as Ag@Fe_3_O_4_, Fe_3_O_4_@TiO_2_, and CuO@Fe_3_O_4_ [120,121].

**Figure 7:**
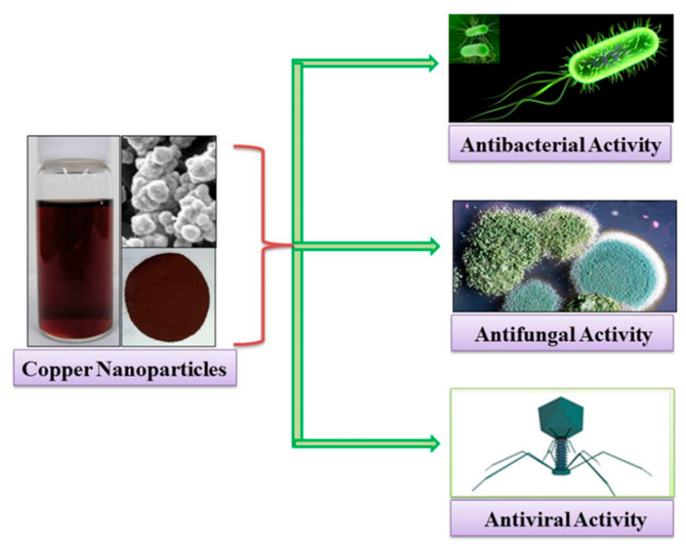
Schematic representation of antimicrobial activity of copper nanoparticles. Reproduced with permission [123].

Das et al. found through the Kirby–Bauer diffusion method that copper nanoparticles could effectively inhibit the growth of *Staphylococcus aureus*, *Bacillus subtilis*, and *Escherichia coli*. Gopalakrishnan et al. proposed a possible mechanism for the mode of action of copper oxide nanoparticles against *Escherichia coli*. The copper oxide nanoparticles adsorbed on the cell surface interact with the cell wall and subsequently cause damage to the cell membrane, increasing its permeability and reducing bacterial viability in copper oxide solution [122]. Chang et al. explained the toxic effects of copper and zinc oxide nanoparticles on eukaryotic cells. Because of their small size, nanoparticles can diffuse directly into cells through pores in the cell membrane or enter cells through ion channels and transporters in the cell membrane. Nanoparticles that enter cells can interact directly with oxidizing organelles such as mitochondria. Subsequently, redox-active proteins stimulate cells to produce reactive oxygen species (ROS), which are induced by ions (Cu^2+^) produced by nanoparticles through various chemical reactions. ROS can cause DNA strand breaks and affect gene expression. In addition, Cu^2+^ ions can form chelates with biomolecules or free metal ions from specific metalloproteins, leading to cytotoxicity [123]. The schematic representation of the antimicrobial activity of copper nanoparticles against bacteria, fungi, and viruses is shown in Figure 7.

In addition, there are magnesium oxide nanoparticles (MgONPs). One of the antibacterial mechanisms of MgONPs is that MgONPs induce oxidative stress in bacterial cells and damage membrane integrity [124]. Another antibacterial mechanism that Leung found through three control experiments is that MgONPs could still produce antibacterial activity against *Escherichia coli* even though MgONPs did not produce reactive oxygen species. After performing characterization tests, Leung et al. suggested that the antibacterial effect was achieved by adsorbing the phosphate groups from the cell surface on the MgO surface [125].

### 4.2. Antibiotic-Loaded Antibacterial Hydrogels

Antibiotics are commonly used to treat bacterial infections. A three-dimensional network porous structure enables the hydrogels to be used as delivery systems for loading antibacterial drugs [126]. Antibacterial hydrogels can release drugs locally in small quantities, reduce bacterial resistance to antibiotics, and provide sufficient bacteria-killing doses for a long time [127]. The antibiotics commonly loaded in antibacterial hydrogels can be divided into aminoglycosides (gentamicin and streptomycin), glycopeptides (vancomycin), and quinolones (ciprofloxacin and levofloxacin) [128]. The main bactericidal mechanisms of conventional antibiotics are as follows: glycopeptide antibiotics inhibit the synthesis of the bacterial cell walls; aminoglycoside antibiotics block protein synthesis pathways; and quinolone antibiotics inhibit the synthesis of nucleic acids [129].

#### 4.2.1. Aminoglycoside-Loaded Antibacterial Hydrogels

Aminoglycoside antibiotics are widely used and effective broad-spectrum antibacterial drugs. Aminoglycosides can destroy protein synthesis through transcellular plasma membrane transport and binding with bacterial ribosomes, thus playing an antibacterial function. Hu et al. prepared a hydrogel with high antibacterial activity against both aerobic and anaerobic pathogens by using crosslinking oxidized dextran with tobramycin and G1-orni. This injectable hydrogel shows thixotropic, biocompatible, self-healing, and pH-responsive drug release behavior. The study provides a facile strategy to expand the antibacterial spectrum of aminoglycoside hydrogels [130]. Li et al. prepared aminoglycosides (AGs) hydrogels based on dynamic covalent bond crosslinking by using AGs, aldehyde hyaluronic acid (A-HA), and adipic acid dihydrazide graft hyaluronic acid (HA-ADH) as materials. The results showed that the hydrogels had excellent and sustained antibacterial properties against *Escherichia coli* and *Staphylococcus aureus*, and A-HA/HA-ADH/AGs hydrogels are potential dressings for wound healing [131].

Gentamicin and streptomycin are aminoglycoside antibiotics, and gentamicin is more commonly used. Gentamicin mainly acts on 30S ribosome 16S rRNA in the bacterial body through three steps: blocking bacterial protein synthesis, destroying the integrity of cell membrane, and leading to cell death. Firstly, gentamicin contains a large number of positively charged amino groups, which combine with the negative charge on the surface of the bacterial membrane, the electrostatic interaction increases the permeability of the bacterial surface, and part of gentamicin molecules penetrate the bacterial interior. Secondly, gentamicin in the cell produces the antibiotic effect, destroys the integrity of the cytoplasmic membrane, and the gentamicin molecule enters the cell in large quantities. Finally, the intracellular gentamicin molecule binds to the A site of the bacterium’s 30S ribosome 16S rRNA, resulting in protein mistranslation and, ultimately, cell death [132]. Guo successfully prepared a series of new crosslinking chitosan quaternary ammonium salt loaded with gentamicin sulfate (CTMCSG) hydrogel membranes by the reaction of chitosan quaternary ammonium salt (TMCS) and epichlorohydrin. The results indicate that the CTMCSG-4 hydrogel film with an attractive physicochemical property, admirable antibacterial activity, and the slight cytotoxicity shows the potential value as an excellent antibacterial wound dressing [133]. Gupta and Purwar prepared hydrogel composites of poly(acrylamide-co-acrylic acid) hydrogel grafted over the cotton fabric using crosslinkers polyethylene glycol (PEG) and *N*,*N*′-methylene bisacrylamide (MBA) separately. The release kinetics of gentamicin sulfate in two kinds of composites were studied, and the results show that the hydrogel composite has higher mechanical strength than its film [134].

#### 4.2.2. Glycopeptides-Loaded Antibacterial Hydrogels

Vancomycin is a typical glycopeptide antibiotic. The mechanism of vancomycin is to inhibit the synthesis of bacterial cell walls and combine with the bacterial cell walls, so that some amino acids cannot enter the glycopeptide of the cell wall. Liao et al. incorporated vancomycin into oxidized hyaluronic acid (HA) and adipic acid dihydrazide, then evaluated the drug release and antimicrobial activity against methicillin-resistant Staphylococcus aureus. The results showed that oxi-HA/ADH hydrogel-loaded with vancomycin has excellent biocompatibility, complete and rapid drug release, and measurable in vitro antimicrobial activity and is quite effective in a biofilm model to prevent immediate bacterial colonization [135]. Naeimi et al. studied a drug-loaded porous hydrogel for the delivery of vancomycin. The hydrogels based on chitosan (CS), Polyvinyl alcohol (PVA), and Polyethylene glycol (PEG) were prepared by lyophilization. The porous CS/PVA/PEG hydrogels containing vancomycin could be good potential candidates as wound dressing [136].

#### 4.2.3. Quinolones-Loaded Antibacterial Hydrogels

**Figure 8:**
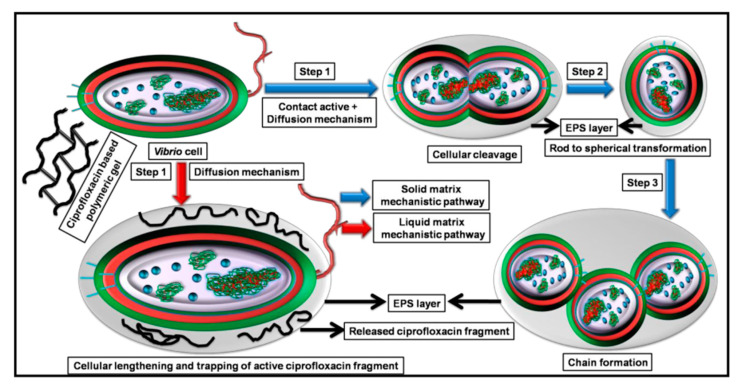
The bactericidal mechanism of DCHBSP gel (ciprofloxacin based polymeric gel) against *V. chemaguriensis* strains in solid and liquid matrices. Reproduced with permission [138].

Quinolone antibiotics mainly include ciprofloxacin and levofloxacin. Among them, ciprofloxacin has an antibacterial effect on broad-spectrum Gram-positive bacteria and Gram-negative bacteria. The antibacterial mechanism of ciprofloxacin relies on the blocking of bacterial DNA replication by binding itself to the DNA rotating-enzyme, which causes bacterial chromosome double-strand breaks, so bacterial resistance to this drug develops slowly [137]. Mukherjee developed an amphoteric hydrogel loaded with ciprofloxacin antibiotics. Ciprofloxacin was covalently linked to a polymer system consisting of hydrophilic polyethylene glycol methyl ether methacrylate (PEGMA) and sulfobetaine methacrylate (SBMA). Ciprofloxacin attached to polymers enables the preparation of highly potent polymeric antibiotic conjugations (PACs) with linear and hyperbranched structures. Its bactericidal mechanism is shown in Figure 8. PACs showed antibacterial activity against both solid and liquid substrates of the strain. After that, through the continuous removal of thiol fragments at the end of ciprofloxacin and the balance of swelling and deswelling process, the hydrogel is transformed into polymeric gel through the “double” contact activity release mechanism, which makes the hydrogel show more potent antibacterial activity than the liquid matrix and achieve a complete bactericidal effect [138].

### 4.3. Hydrogels with Inherent Antibacterial Activity

#### 4.3.1. Antimicrobial Peptide Hydrogel

**Figure 9:**
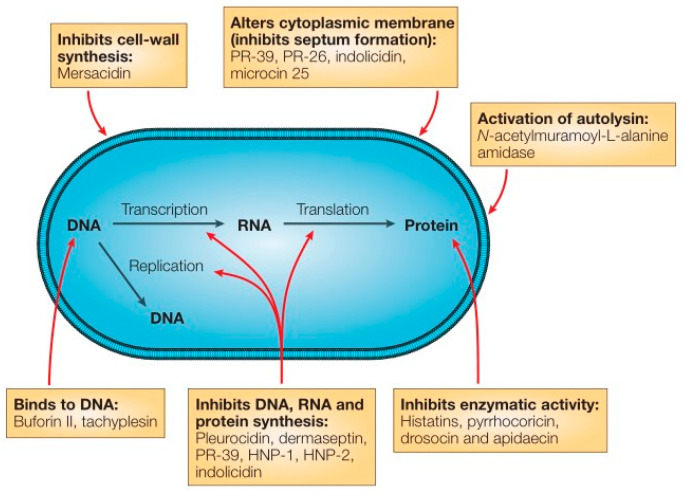
Mode of action for intracellular antimicrobial peptide activity. Reproduced with permission [149].

Antimicrobial peptides (AMPs) have many antibacterial activities and exist widely in many animal and plant tissues and cells. AMPs are strongly alkaline, have thermal stability, and have vigorous antibacterial activity against microorganisms such as bacteria, fungi, and viruses [139,140]. As a new type of non-antibiotic antibacterial agent, antimicrobial peptides are considered the most likely drugs to replace antibiotics [141,142,143]. At present, many studies believe that the antimicrobial mechanism of AMPs is that antimicrobial peptides are adsorbed on the lipid membrane through static electricity, which destroys the internal and external barrier of bacterial cells, leading to the increase of membrane permeability and the death of bacteria due to the inability to maintain normal osmotic pressure. This mechanism of action is less likely to lead to bacterial resistance [144,145,146]. Some studies believe that antimicrobial peptides inhibit bacterial metabolic behavior by inhibiting bacterial membrane synthesis, DNA synthesis, and enzyme activity, thus leading to bacterial inactivation or death [147,148]. The antibacterial mechanism of antimicrobial peptides is shown in Figure 9. In this figure, *Escherichia coli* is shown as the target microorganisms.

In recent years, many antibacterial hydrogels with antibacterial peptides have been developed. Aye et al. use a simple method based on electrostatic interaction to prepare antibacterial silk hydrogels containing antimicrobial peptides (AMP) [150]. Baral et al. prepared an antibacterial dipeptide, which showed excellent antibacterial activity against G− bacteria (*E. coli* and *P. aeruginosa*), as well as high biocompatibility with human red blood cells and human fibroblast cells [151]. Zhou et al. reported that a popular AMP polylysine had been applied in photopolymerized antibacterial hydrogels, which generated good coatings for medical devices and implants [152]. Bai et al. designed an amphiphilic peptide A_9_K_2_ that could effectively inhibit both G+ and G− bacterial strains [153]. To produce antibacterial hydrogel composite wound dressing, Gunes and Ziylan mixed carboxymethyl chitosan (CMCht)-sodium alginate-streptococcus lactic into cotton and modified it without damaging its beneficial properties. The modified wound dressing retained the porous structure and produced adequate WVTR [154]. Atefyekta et al. studied antimicrobial peptide-functionalized mesoporous hydrogels; amp-hydrogel has high antibacterial activity against *Staphylococcus epidermidis*, *Staphylococcus aureus*, *Pseudomonas aeruginosa*, methicillin-resistant *Staphylococcus aureus* (MRSA), and multidrug-resistant *Escherichia coli*, and has potential hemostatic activity [155].

#### 4.3.2. Chitosan Antibacterial Hydrogel

Chitosan is a kind of typical antibacterial cationic polymer. The amino groups in its molecular chain can be protonated in an acidic environment and have positive electric properties [156]. The modification of chitosan molecules can improve its water solubility, and its derivatives, such as chitosan quaternary ammonium salt, have good solubility in neutral pH, which significantly enhances its antibacterial activity in a neutral environment. Introducing chitosan and its derivatives has become a standard method to imbibe materials with inherent antibacterial properties. Hu et al. used Ca^2+^ as a physical crosslinking agent to design a natural polysaccharide hydrogel with double physical crosslinking. Cationic polymer chitosan (CS) forms ionic coordination with polyanionic polymer sodium alginate (SA), and Ca^2+^ forms ionic coordination with the carboxyl group on the SA molecular chain. The polysaccharide hydrogel synthesized by this method can be a suitable carrier of epidermal growth factors to promote cell proliferation and wound healing [157]. Yu et al. used positively charged CS and negatively charged carrageenan (κ-CG) to prepare a polysaccharide-based hydrogel film with good toughness and anti-cell adhesion properties. In addition to the ionic coordination between the CS and κ-CG molecular chain, hydrogen bonds can also form between the CS and κ-CG molecular chain. Through the synergistic effect of ionic coordination and hydrogen bonds, the hydrogel film has excellent self-healing properties, and the self-healing efficiency can reach 90% after the initial stretching and standing for two h [158]. Wang reported a biodegradable chitosan hydrogel system with excellent injectable, antibacterial activity, biocompatibility, and self-repairing properties. The primary raw materials for the hydrogel preparation are chitosan and aldehyde chitosan. Due to the antibacterial activity of chitosan and aldehyde chitosan in the hydrogel network, the hydrogel system can significantly inhibit the growth of bacteria upon contact, making it an excellent candidate for antibacterial wound dressing [159]. Zhang et al. developed a new antibacterial hydrogel wound dressing composed of poly(aminoethyl) modified chitosan (PAEMCS). Experiments showed that the hydrogel based on PAEMCS had excellent hygroscopicity; antibacterial activity against *Escherichia coli*, *Staphylococcus aureus*, and *Staphylococcus epidermidis*; and good cytocompatibility against L929 cells or HUVEC [160]. Chen et al. immobilized biodegradable antibacterial polymer chitosan (CS) onto the surface of poly(N-isopropyl acrylamide) (PNIPAAm)gel/polypropylene(PP) nonwoven composites for wound dressing applications. The results showed that the product had the antibacterial ability to *Escherichia coli* and *Staphylococcus aureus* and had good biocompatibility with fibroblasts [161].

#### 4.3.3. Amphoteric Ion Antibacterial Hydrogel

The antibacterial mechanism of amphoteric ionic hydrogels is similar to that of AMPs. The molecular chain of cationic polymers contains many positively charged groups. The positively charged polymer molecular chain is bound to the negatively charged bacterial cell membrane through ion coordination, forming a polymer film on the bacterial surface and hindering the transport of internal and external substances. The structure of the bacterial cell membrane is destroyed, which affects the osmotic balance of bacteria inside and outside and causes the outflow of the intracellular matrix, resulting in bacterial death [162,163]. Panini et al. prepared a new type of poly hydroxyethyl methacrylate-PAM/PVA/CS interpenetrating polymer network (IPN) hydrogel by two-step radical polymerization. CS and polycations interact with negatively charged surfaces of bacteria, resulting in membrane loss of permeability, cell leakage, and eventual death. The antibacterial hydrogel has higher swelling, mechanical strength, and antibacterial activity than chitosan, and has good antibacterial activity against Gram-negative bacteria. It is an ideal material for preparing low-cost wound dressings [164]. Wang et al. designed and introduced a novel structure of amphoteric ionic poly[3-(dimethyl (4-vinyl benzyl) ammonium) propyl sulfonate] (SVBA) into the polyAAm network to obtain a novel salt-responsive conductive hydrogel. Due to the addition of SVBA, PAS-2 hydrogel has good electrical activity and significant mechanical properties, as well as good cellular compatibility, stable rheological properties, effective antibacterial properties, strong adsorption, and high permeability, which can effectively promote wound healing and can be used as a medical dressing [165]. Dai et al. developed zwitterionic sulfobetaine acrylamide antibacterial hydrogel combined with laponite (LAP) nanoplatelets and methyl acrylamide dopamine (DMA) for wound dressings. LAP nanoplatelets and DMA impart the hydrogel-enhanced mechanical strength and material adhesion, respectively. The multifunctional hydrogel dressing with antifouling, antibacterial properties, and proper adhesion was successfully constructed without secondary damage to the repaired tissues [166]. Zhu et al. developed a new type of zwitterionic hydrogel, polycarboxy betaine (PCB) hydrogel, as a wet dressing to promote skin wound healing. The high water content may promote autolysis debridement, stimulate collagen deposition, and accelerate wound healing [167].

### 4.4. Photosensitive Antibacterial Hydrogel

Photodynamic therapy (PDT) is another effective antibacterial method. Under the influence of light of an appropriate wavelength, a photosensitive agent (PS) is stimulated to generate reactive oxygen species or singlet oxygen to destroy bacteria, resulting in the rupture of the bacterial cell membrane and the destruction and inactivation of protein structure, thus achieving the purpose of sterilization [168,169,170]. Fighting bacterial infections in this way does not lead to the emergence of resistant bacteria. The combination of photosensitizer hydrogel is an effective therapeutic strategy for the treatment of pathogen infection [171]. Three cationic photosensitizers, (Meso-tetra (4-*N*-methylpyridyl) porphyrinetetratosylate (TMPyP), Zn (II) meso-tetra (*N*-methyl-4-pyridyl) porphinetetra chloride (Zn-TMPyP), and methylene blue (MB)), were adsorbed by montmorillonite (MMT). The photosensitive MMT/PS hydrogels were prepared by UV crosslinking method in an aqueous solution of polyvinyl alcohol-styrylpyridine salt condensation (PVA-SbQ). The results showed that all three photosensitive MMT/PS hydrogels had good swelling performance and a specific killing effect on Staphylococcus aureus [172]. Truong and Forsythe studied photolabile hydrogels and found that nanocrystalline TiO_2_ can be used as a photoinitiator of polymer PEGDA hydrogel. TiO_2_ is evenly distributed in the matrix and still has photoactivity under fluorescent lamp irradiation. It can be used as a photoinitiator in the hydrogel polymerization process and as a photosensitizer in photodynamic therapy [173]. Deng et al. prepared agarose (AG)-based hydrogel containing tannic acid-Fe(III) (TA-Fe) nanoparticles by a facile and eco-friendly strategy, which has good mechanical properties, excellent photothermal effect, and good biocompatibility. And photothermal can effectively avoid the disadvantages of traditional antibiotics in killing pathogenic bacteria and treating wound infection, and it is expected to be an antibacterial wound dressing in the biomedical field [174]. Zhou Qian separated the photosensitive antibacterial agent from the lotus, using tryptophan to modify the natural photosensitive agent tribithiophenal, improve water solubility at the same time, and reduce the phototoxicity. The modified photosensitizer was filled into the blisters, and the resulting blisters were loaded on the dressing. Photosensitive intelligent antibacterial hydrogel dressing was obtained [175].

### 4.5. Hydrogels with Synergetic Effects

Synergistic antibacterial hydrogels refer to antibacterial hydrogels with synergistic action of two or more antibacterial modes to improve antibacterial performance [176]. There are many reports on antibacterial hydrogel products that enhance the antibacterial effect by using silver nanoparticles synergistic with antibiotics or graphene oxide (GO).

**Figure 10:**
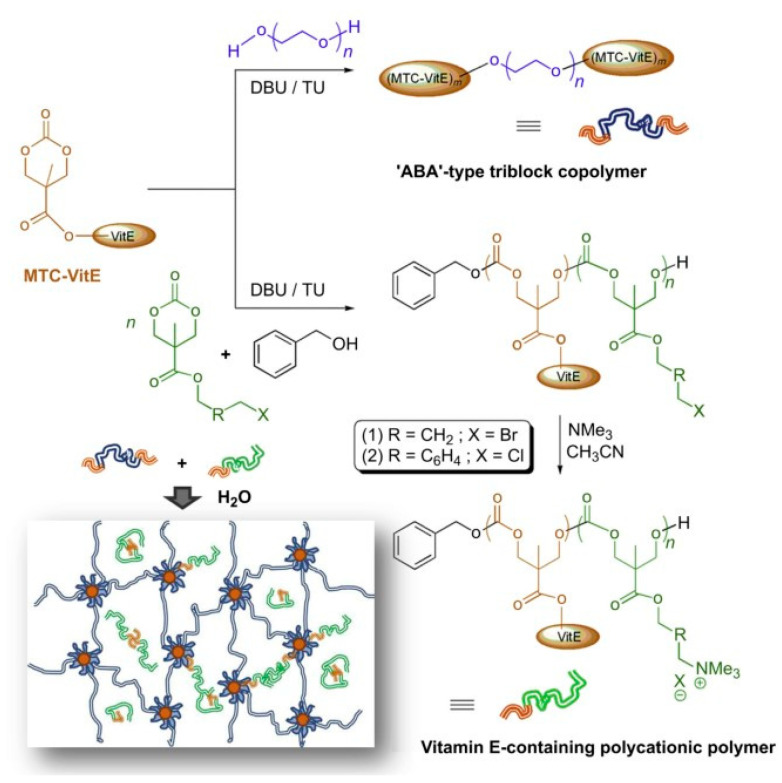
Syntheses of ‘(MTC-VE)n-PEG-(MTC-VE)n’ and vitamin E-containing polycationic polymers. Schematic illustration of incorporating polycationic polymers into hydrogel system (inset). Reproduced with permission [179].

Bu studied the preparation and antibacterial properties of polymer-modified graphene oxide and its hydrogel composites; the modified GO hydrogel wound dressing was prepared by combining GO, quaternary ammonium salt (QAS), and N-halamine. The sauce has good biocompatibility and excellent antibacterial effects, promoting wound healing [177]. The nanofiber felt was prepared by blending chitosan-based silver nanoparticles with polyvinyl alcohol. Then, the nanofibers with an average diameter of 150 nm were prepared by crosslinking with glutaraldehyde and electrospinning. The results showed that the combination of chitosan and Ag-NPS had an excellent synergistic antibacterial effect. The presence of Ag NPs in the PVA/CS blend solution not only enhanced the electrospinning performance, but also enhanced the antibacterial ability of electrospinning felt, which is a good wound dressing [178]. Lee et al. developed a biodegradable antibacterial hydrogel using vitamin E functionalized polycarbonate and achieved a better synergistic antibacterial effect by combining antibacterial polycarbonate with the conventional antifungal agent fluconazole. Syntheses of the polymers and the schematic illustration of incorporating polycationic polymers into the hydrogel system are shown in Figure 10.

Yang studied the preparation and properties of antibacterial hydrogel based on polyvinyl alcohol [180]. Palantoken et al. studied UV-cured cationic quaternary ammonium polyethylenimine and embedded it with silver nitrate to prepare hydrogels with dual bacteriostatic effects [181]. Mao et al. studied the photo-inspired antibacterial activity of Ag/Ag@AgCl/ZnO nanostructured hydrogels. The hydrogel has high antibacterial efficiency against both *Escherichia coli* and *Staphylococcus aureus*. The release of Ag^+^ and Zn^2+^ stimulates the immune function to produce many white blood cells and neutrophils, thereby producing synergistic antibacterial effects and accelerated wound healing [182]. Yang et al. developed a slow-release antibacterial hydrogel based on branched polyethyleneimine (PEI), stabilized GO-AgNPs, and Pluronic F127 for a more stable and sustained antibacterial effect [183,184]. Kang et al. prepared a temperature-sensitive antibacterial hydrogel NIPOM-CG/GM with antibacterial properties based on vinyl carboxymethyl chitosan (CG), silane dispersed graphene (GM), and N-isopro polyacrylamide (NIPAM) [185].

## 5. Summary and Prospect

Because of its good biocompatibility and moisture absorption, traditional hydrogels have gradually become common materials in people’s daily lives. Especially in the medical field, hydrogels with antibacterial functions play an important role. They are especially suitable for biomedical applications such as wound dressing, contact lenses, and controlled release of drugs. The abuse of antibiotics in medical treatment will lead to bacterial resistance, and some traditional hydrogels are gradually withdrawn from the market. To have new biological functions, more intelligent antibacterial hydrogels need to be designed. With further research, the types of antibacterial hydrogels are gradually enriched. In this paper, according to different antibacterial modes, antibacterial hydrogels can be divided into five types: antibacterial hydrogel containing inorganic nanoparticles; antibiotic-loaded antibacterial hydrogels; hydrogels with inherent antimicrobial activity; photosensitive antibacterial hydrogels; and hydrogels with synergetic effects. The mechanism, properties, and preparation methods of these antibacterial hydrogels also expound. Experimental research shows that some antibacterial agents can also be synergies with other components; in addition, antibacterial hydrogel has a better antibacterial effect and a broader antibacterial spectrum. It also provides a diverse performance, such as water/moisture absorption, slow-release drug properties, swelling properties, permeability, ultraviolet resistance, etc. Graphene oxide (GO) and nanosilver (Ag-NPs) are considered promising antibacterial materials due to their excellent biocompatibility, broad-spectrum antibacterial properties, and non-drug resistance. Antimicrobial peptides and cationic polymers have also been used to synthesize inherently antibacterial hydrogels that effectively treat bacterial infections. The modified GO hydrogel has an excellent antibacterial effect and can promote wound healing. The combination of chitosan and Ag-NPS has uniquely promising properties and a synergistic antibacterial effect. Of course, as a wound dressing, the antibacterial hydrogel will develop into an intelligent and multi-functional tool in the future and solve the problems of mechanical strength, adhesion performance, and permeability.

## Data Availability

Not applicable.
